# Impact of facial profile on young adults’ oral health-related quality-of-life item levels: A hierarchical analysis

**DOI:** 10.1590/2177-6709.26.6.e2120147.oar

**Published:** 2021-12-15

**Authors:** Marcelo Venturinelli MARTINS, Patrícia Rafaela dos SANTOS, Diego Patrik Alves CARNEIRO, Marcelo de Castro MENEGHIM, Carolina Carmo de MENEZES, Silvia Amélia Scudeler VEDOVELLO

**Affiliations:** 1 Centro Universitário da Fundação Hermínio Ometto, Departamento de Ortodontia (Araras/SP, Brazil).; 2 Universidade Estadual de Campinas, Faculdade de Odontologia de Piracicaba (Piracicaba/SP, Brazil).

**Keywords:** Oral health related quality of life, Malocclusion, Orthodontic treatment need, Facial profile

## Abstract

**Objective:**

To assess the impact of facial profile on young adults’ oral health-related quality of life (OHRQoL) item levels.

**Methods:**

A cross-sectional study was carried out with a population-based sample of 205 young adults, with a mean age of 23.1 years. The individuals answered questions about OHRQoL (OHIP-14) and self-esteem (Global Negative Self-Evaluation). The Dental Health Component (DHC) of the Index of Orthodontic Treatment Need (IOTN) was used to evaluate normative orthodontic treatment needs and define dental malocclusion clinically. Facial profile was analyzed using photographs and dichotomized into two levels: normal (straight) and altered facial profile (convex or concave). A calibrated researcher performed the clinical examination. Association between the independent variables and the outcome (OHRQoL) was established by hierarchical multiple linear regression analysis for each item level. Considering the variable of interest (facial profile), the psychological incapacity domain was the most affected item.

**Results:**

Individuals with changed facial profile had 2.47 (1.04-5.85) times higher chances of reporting impacts on psychological incapacity than those with a normal profile (*p*> 0.05). The association was modulated by dental malocclusion and self-esteem.

**Conclusions:**

The convex and concave facial profile showed a negative impact on the psychological aspects of young adults’ quality of life.

## RESUMO

**Objetivo:**

Avaliar o impacto do perfil facial nos domínios da qualidade de vida relacionada à saúde bucal (QVRSB) de jovens adultos.

**Métodos:**

Estudo observacional transversal realizado com 205 jovens adultos, com idade média de 23,1 anos. Os indivíduos responderam questões sobre QVRSB (OHIP-14) e autoestima (*Global Negative Self-Evaluation*). O Componente de Saúde Bucal (DHC) do Índice de Necessidade de Tratamento Ortodôntico (IOTN) avaliou as necessidades normativas de tratamento ortodôntico e definiu clinicamente a má oclusão dentária. O perfil facial foi analisado por meio de fotografias e dicotomizado em dois níveis: perfil facial normal (reto) e perfil facial alterado (convexo ou côncavo). Um pesquisador calibrado realizou os exames clínicos. A associação entre as variáveis independentes e o desfecho (QVRSB) foi determinada pela análise de regressão linear múltipla hierarquizada para cada domínio do OHIP-14. Considerando a variável de interesse (perfil facial), o domínio incapacidade psicológica foi o item mais afetado.

**Resultados:**

Indivíduos com perfil facial alterado tiveram 2,47 (1,04-5,85) vezes mais chances de relatar impacto na incapacidade psicológica do que aqueles com perfil normal (*p* > 0,05). A associação foi modulada pela má oclusão dentária e a autoestima.

**Conclusões:**

O perfil facial alterado (convexo ou côncavo) apresentou impacto negativo nos aspectos psicológicos da qualidade de vida relacionada à saúde bucal de adultos jovens.

## INTRODUCTION

Clinical orthodontic diagnosis frequently ignores the psychosocial conditions perceived by individuals.^1^ Although the normative evaluation is essential, self-perception provides important information about the impact of malocclusion on an individual’s life.^1^ ^-^ ^3^ In this context, self-perception is related to psychosocial well-being and may impact the quality of life.^1^ ^,^ ^3^ ^,^ ^4^ Furthermore, individuals with a negative perception of their esthetic appearance have lower self-esteem^2^ ^,^ ^3^ and lower oral health-related quality of life (OHRQoL)^4^ ^-^ ^6^ than those who consider themselves attractive. Self-esteem is determined by a set of factors including occlusal balance and an attractive facial profile.^2^ ^,^ ^3^ However, the studies carried out to date are based on dental aesthetics and generally do not assess the impact of the facial profile in this context.

The impact of malocclusion on the OHRQoL is considered a controversial topic because some studies have confirmed^7^ ^,^ ^8^ and others have denied association between them.^3^ Nevertheless, it should be highlighted that malocclusion has been evaluated based on occlusion indicators. If we consider that the desire for a better physical appearance is the reason most frequently reported by individuals seeking orthodontic treatment,^3^ ^,^ ^8^ ^-^ ^10^ the face must be considered a significant predictor of patients’ expectations of treatment outcomes. However, there are no studies about the impact of skeletal changes reflected in the facial profile on young adults’ OHRQoL. In addition, according to the literature,^11^ because studies reporting item levels comparisons between OHRQoL and malocclusion are scarce, which physical and psychological health items have a stronger influence on orthodontic treatment needs remains unclear, and generalizations can be made based only on limited studies. 

Thus, considering the hypothesis that the face is an important factor in the evaluation of aesthetic concern, this study aimed to evaluate the impact of facial profile on young adults’ OHRQoL item levels. The assessment was performed on the item level analysis of OHRQoL.

## MATERIAL AND METHODS

### STUDY DESIGN, PARTICIPANTS, AND SAMPLE SIZE

A population-based cross-sectional study was conducted involving 205 young adults. The minimum sample was calculated assuming a test power of 80%, level of significance of 5%, and an effect size of 1.8. The finite population was used by considering prevalence of 50% of OHRQoL.^3^ ^,^ ^4^ The sample included young Brazilian adults of both sexes, aged between 18 and 35 years, with an average age of 23.1 years (SD 1.02). The study included only white individuals, due to differences in facial profile between ethnicities. The evaluation was carried out by the investigator. Current or previous orthodontic treatment, systemic diseases, cleft lip, and/or palate syndromes were exclusion criteria. Data collection was performed between August and November 2018. This study received approval from the Human Research Ethics Committee (*Centro Universitário Hermínio Ometto*, #74585417.3.0000.5385).

### STUDY INSTRUMENTS

The questionnaires were self-administered at the time of data collection. The individuals answered questions about OHRQoL and self-esteem. 

The outcome variable was OHRQoL at an item level. The Brazilian version of Oral Health Impact Profile (OHIP-14),^12^ ^,^ ^13^ was used to evaluate the impact on OHRQoL. The OHIP-14 comprises 14 questions in seven domains with two items each: (1) functional limitation; (2) physical pain; (3) psychological discomfort; (4) physical incapacity; (5) psychological incapacity; (6) social incapacity, and (7) social disadvantage. Each response received a score: 0 corresponded to never, 1 = rarely, 2 = sometimes, 3 = repeatedly, and 4 = always. The questionnaire score was obtained by the sum of scores, and could vary from 0 to 56, with higher scores indicating negative impacts on quality of life. The result of each item level was dichotomized by the median. A score of 2 or less on two questions of each item level indicated absence of impact, and a score higher than 2 indicated an impact on the quality of life.^4^

The Global Negative Self-Evaluation (GSE)^14^ was used to evaluate self-esteem. The GSE consists of a scale with six items; each item has six possible responses that are quantified in increasing order (1 to 6), according to their disposition in the scale. Thus, to rank self-esteem, the sum of all the responses is divided by six to obtain the value of individual self-esteem in four categories: 1-1.69, very little negative self-assessment; 1.7-2.69, little negative self-assessment; 2.7-3.99, some negative self-assessment; 4.0-6.0, very negative self-assessment. The individuals were ranked as having high (values < 2.69) or low (values > 2.7) self-esteem.^2^

### FACIAL AND CLINICAL MEASURES

All participants had standardized clinical examinations, including intraoral occlusal measurements and clinical photographs.

Dental malocclusion was evaluated by means of the Dental Health Component of the Index of Orthodontic Treatment Need (IOTN-DHC).^15^ The clinical exam was performed with a disposable lip retractor with the patient in a seated position in a room with natural lighting. By means of a scale of five grades in ascending order, the IOTN-DHC is used to assess: crowding, missing teeth (including congenital absence and impacted teeth), overjet (positive or negative), anterior or posterior crossbite, overbite, and anterior or posterior open bite. All the conditions were evaluated, and only the most severe were used as a basis for determining treatment need. For data analysis, normative orthodontic treatment need was dichotomized into IOTN-DHC grades: grades 1 to 2, without dental malocclusion or orthodontic treatment need; and grades 3 to 5, with malocclusion and orthodontic treatment need.^15^

Facial profile photographs were obtained in a standardized way, considering the distance between each volunteer and the camera. The photos were taken using a SLR D7000 camera (Nikon do Brazil Ltda.), with Nikon 18-200 mm VR f/3.5-5.6G II lens (Nikon do Brazil Ltda).^16^ The camera was positioned parallel to the ground on a leveled tripod. The individuals sat on a chair next to a white wall and were instructed to look straight ahead at a horizontal line in the natural position of the head^17^, and then the profile photograph was taken. This procedure was adopted for all the study participants. In the photographs, an angle of convexity of the facial profile (G.Sn-Pog’, G = Glabella point; Sn = Subnasal point; Pog’ = Soft tissue pogonion point) was traced using Photoshop software (CS 8.0.1; Adobe Systems, San Jose, California). The individuals were classified as having straight, convex, or concave profiles, according to the soft tissue analysis.^18^ ^-^ ^21^ A convexity angle of 8-16° indicated a straight profile, while an increase or decrease of the angle indicated a convex or concave profile, respectively.^18^ ^-^ ^21^ As a methodological criterion, individuals were classified into two categories: with a normal facial profile (straight profile) or with an altered facial profile (convex or concave profile).^20^ ^,^ ^22^

### CALIBRATION

The clinical oral examination was performed by one researcher who was properly calibrated and had epidemiological experience and orthodontic knowledge. The consistency of intra-examiner agreement was assessed by weighted Kappa, obtaining a value of 0.94. The method error for assessing facial profiles was verified in 30% of the sample using random selection after a 30-day interval. The random errors were calculated according to Dahlberg’s formula^23^ and the systematic errors were evaluated with dependent *t*-tests (*p*< 0.05) to allow verification of the absence of significant difference. 

### STATISTICAL ANALYSIS

**Figure 1:**
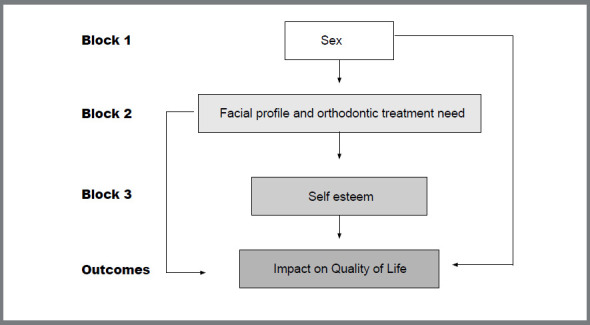
Hierarchical multiple logistic models flowchart.

The data were analyzed initially by frequency distribution tables. Simple logistic regression models were constructed for the independent variables and each item level of the OHIP-14 as the outcome. Subsequently, hierarchical multiple logistic models were estimated considering the following hierarchy: block 1 (sex), block 2 (orthodontic treatment need and facial profile), and block 3 (self-esteem), according to Figure 1. 

The variables with *p*< 0.20 in each block were tested in multiple regression models, and those with *p*≤ 0.10 remained in the model after adjustment for the variables in the same block and previous block. By means of the regression models, the raw and adjusted odds ratios were estimated with confidence intervals of 95%. Analyses were performed with the R software (R Foundation for Statistical Computing, Vienna, Austria).

## RESULTS

The population of this study was composed of 205 young adults with a mean age (±SD) of 23.1 ± 1.02 years. Table 1 shows the frequency of distribution relative to OHIP-14 item levels, considering the variables analyzed. Higher frequencies were observed in the physical pain and psychological discomfort domains.

**Table 1:** Individuals with impact on oral health-related quality-of-life item levels considering the studied characteristics.

Variable	Category	n (%)	With OHRQoL impact						
			Functional limitation	Physical pain	Psychological Discomfort	Physical Incapacity	Psychological Incapacity	Social Incapacity	Social Disadvantage
				n (%)	n (%)	n (%)	n (%)	n (%)	n (%)
Sex	Female	75 (36.6)	2 (2.7)	23 (30.7)	20 (26.7)	5 (6.7)	9 (12.0)	6 (8.0)	3 (4.0)
	Male	130 (63.4)	8 (6.2)	53 (40.8)	57 (43.8)	12 (9.2)	25 (19.2)	5 (3.8)	2 (1.5)
Facial profile*	Straight	83 (40.5)	1 (1.2)	33 (39.8)	25 (30.5)	3 (3.6)	8 (9.6)	1 (1.2)	1 (1.2)
	Concave/Convex	122 (59.5)	9 (7.4)	43 (35.2)	52 (42.6)	14 (11.5)	26 (21.3)	10 (8.2)	4 (3.3)
Dental malocclusion**	Without	163 (79.5)	5 (3.1)	56 (34.4)	58 (35.6)	10 (6.1)	24 (14.7)	6 (3.7)	3 (1.8)
	With	42 (20.5)	5 (11.9)	20 (47.6)	19 (45.2)	7 (16.7)	10 (23.8)	5 (11.9)	2 (4.8)
Self-esteem	Normal	173 (84.4)	7 (4.0)	57 (33.0)	58 (33.5)	15 (8.7)	23 (13.3)	8 (4.6)	5 (2.9)
	Low	32 (15.6)	3 (9.4)	19 (59.4)	19 (59.4)	2 (6.2)	11 (34.4)	3 (9.4)	0 (0.0)

*Normal (straight) or changed (concave or convex) profile; **IOTN-DHC: 1 to 2, without treatment need; 3 to 5, with orthodontic treatment need.

Table 2 shows the analysis of association between the independent variables and presence of impact on each item level of the OHIP-14. Considering the variable of interest (facial profile), psychological incapacity was the item level most affected. Individuals with facial profile convex/or concave had a 2.47 (1.04-5.85) times higher chance of reporting impacts on psychological incapacity than those with straight profile. 

**Table 2:** Association between the independent variables and presence of impact on oral health- related quality-of-life item levels.

Variable	Category	Dom1		Dom2		Dom3		Dom4		Dom5		Dom6		Dom7	
		Raw OR (CI)	Adjusted OR (CI)	Raw OR (CI)	Adjusted OR (CI)	Raw OR (CI)	Adjusted OR (CI)	Raw OR (CI)	Adjusted OR (CI)	Raw OR (CI)	Adjusted OR (CI)	Raw OR (CI)	Adjusted OR (CI)	Raw OR (CI)	Adjusted OR (CI)
Block 1															
Sex	Female	Ref.	-	Ref.	-	Ref.	Ref.	Ref.	-	Ref.	-	Ref.	-	Ref.	-
	Male	2.39 (0.50-11.58)	-	1.56 (0.85-2.84)	-	2.15 (1.16-3.98)	2.24 (1.19-4.21)	1.42 (0.48-4.21)	-	1.75 (0.77-3.97)	-	0.46 (0.14-1.56)	-	0.38 (0.06-2.30)	-
	p-value	0.2779	-	0.1504	-	0.0154	0.0128	0.5233	-	0.1837	-	0.2132	-	0.2888	-
Block 2															
Facial profile	Straight	Ref.	-	Ref.	-	Ref.	-	Ref.	-	Ref.	Ref.	Ref.	-	Ref.	-
	Concave/ convex	6.53 (0.81-52.55)	-	0.82 (0.46-1.47)	-	1.72 (0.96-3.11)	-	3.46 (0.96-12.43)	-	2.54 (1.09-5.93)	2.47 (1.04-5.85)	7.32 (0.92-58.31)	-	2.78 (0.30-25.32)	-
	p-value	0.0778	-	0.5116	-	0.0708	-	0.0576	-	0.0313	0.0396	0.0601	-	0.3645	-
Dental malocclusion	Without	Ref.	Ref.	Ref.	-	Ref.	-	Ref.	Ref.	Ref.	-	Ref.	-	Ref.	-
	With	4.27 (1,18-15.52)	4.27 (1,18-15.52)	1.74 (0.87-3.45)	-	1.50 (0.75-2.97)	-	3.06 (1.09-8.60)	3.06 (1.09-8.60)	1.81 (0.79-4.16)	-	3.54 (1.02-12.22)	-	2.67 (0.43-16.50)	-
	p-value	0.0274	0.0274	0.1149	-	0.2509	-	0.0338	0.0338	0.1621	-	0.0458	-	0.2915	-
Block 3															
Self-esteem	Normal	Ref.	-	Ref.	Ref.	Ref.	Ref.	Ref.	-	Ref.	Ref.	Ref.	-		-
	Low	2.45 (0.60-10.04)	-	2.97 (1.37-6.44)	2.97 (1.37-6.44)	2.90 (1.34-6.28)	3.04 (1.38-6.69)	0.70 (0.15-3.23)	-	3.42 (1.46-8.00)	3.33 (1.40-7.90)	2.13 (0.53-8.52)	-	-	-
	p-value	0.2117	-	0.0057	0.0057	0.0070	0.0128	0.6498	-	0.0047	0.0065	0.2832	-	-	-

OR = Odds ratio. CI = Confidence Interval. Dom1 = functional limitation; Dom2 = physical pain; Dom3 = psychological discomfort; Dom4 = physical incapacity; Dom5 = psychological incapacity; Dom6 = social incapacity; Dom7 = social disadvantage.

On the other hand, the association was modulated by dental malocclusion and self-esteem. Individuals with dental malocclusion and orthodontic treatment needs had a 4.27 (1.18-15.25) times higher chance of reporting functional limitation and a 3.06 (1.09-8.60) times higher chance of reporting physical incapacity. Individuals with low self-esteem had a 2.97 (1.38-6.44) times higher chance of reporting physical pain, 3.04 (1.38-6.69) times higher chance of reporting psychological discomfort, and 3.33 (1.40-7.90) times higher chance of reporting physiological incapacity. The item levels of social incapacity and social disadvantage were not affected by independent variables. 

## DISCUSSION

The literature has shown the growing importance of analyzing individual perceptions related to orthodontic treatment need.^2^ ^,^ ^6^ ^,^ ^24^ In the present study, clinical treatment need was evaluated by the dental health component (DHC) of the IOTN and defined the dental malocclusion. Although they are similar instruments of evaluation,^25^ the IOTN is more suitable than the Dental Aesthetic Index (DAI) because it considers the functional aspects of occlusion.^26^

The main differential of this study was the inclusion of facial profile analysis in the epidemiological research of malocclusion. Thus, we associate dental analysis (IOTN) of occlusion with the facial profile to understand the impact of possible skeletal problems, suggested by the facial profile, in epidemiology. The facial profiles were classified based on the convexity angle,^18^ ^-^ ^20^ which is indicated for determining the morphology of the soft tissues of the face.^27^ Reference values were considered for the Brazilian population,^21^ and straight, convex, and concave profiles^20^ ^-^ ^22^ were identified to answer the following question: What is the impact of facial profile on adults’ OHRQoL?

Our results showed that individuals with a convex or concave profile were more likely to report psychological impacts on their quality of life. According to the literature, there is a preference for a straight profile that corresponds to a Class I skeletal pattern,^28^ which reflects facial attractiveness.^29^ Thus, the most likely explanation for our results is that the anteroposterior aspect of the face is considered an important factor in the evaluation of aesthetics, which justifies the impact related to convex (Class II pattern) and concave (Class III pattern) profiles. The results supported the importance of in-depth investigation at the item level of OHRQoL assessment scales. In addition, an altered facial morphology may be associated with less self-confident in social relationships, since a severe malocclusion can affect how a person is perceived negatively throughout his or her entire life. Maybe it is another possible explanation for the association between the changed facial profile and the psychological incapacity domain.^30^

In addition, the isolated diagnosis of malocclusion affects the functional limitation and physical incapacity of the individuals. Although some studies report no impact on the OHRQoL,^3^ ^,^ ^27^ the item level analysis showed an association of functional and physical aspects. It is essential to highlight that the need for orthodontic treatment was studied based on the prioritization of dental criteria and that the inclusion of soft tissue analyzes will allow a better understanding of dentoskeletal problems. 

In this sense, self-esteem should not be ignored. The subjective analysis of self-esteem has a direct influence on assessments involving aesthetic concern, and individuals with low self-esteem tended to report impacts on the OHRQoL.^3^ ^,^ ^10^ Thus, our results showed that individuals with low self-esteem reported a negative impact on physical pain and the psychological aspects of OHRQoL. In the same context, the gender variable was associated with a higher chance of impact on psychological discomfort. The literature has affirmed that women report greater oral health-related social and psychological impacts than men.^10^ ^,^ ^29^ ^,^ ^30^ The main difference in the findings concerned the age range; the majority of studies that have observed greater impacts on women^10^ ^,^ ^29^ ^,^ ^30^ evaluated adolescents; in our study, we evaluated young adults. 

Studies that investigated the impact of malocclusion on adults’ OHRQoL were based exclusively on dental indicators, such as DAI and IOTN,^3^ ^,^ ^6^ ^,^ ^7^ or including the cephalic index.^2^ This is the first study to include soft tissue analysis in the observational epidemiological evaluation. We evaluated faces using photographs, which provided reliability for the facial profile diagnosis. Perhaps this is the controversy in the literature; the fact that the anteroposterior skeletal pattern has not been included in the OHRQoL evaluation. Individuals with normal occlusion do not necessarily have a Class I skeletal pattern. Moreover, the orthodontic clinic frequently observes individuals with skeletal deformities, who report that malocclusion affects their daily lives. Thus, the strength of this study was to include the facial profile among the possible clinical aspects that affect OHRQoL. 

The cross-sectional design of the study may be considered a limitation because the impact of dental and facial changes was evaluated at a specific time. A longitudinal study design would also strengthen the study and possibly deepen understanding of the intensity and extent to which these aspects cause in the individual’s life.

Finally, the findings do not support the study hypothesis; in both the clinical and epidemiological context, there is a need for an approach to evaluating individuals’ perceptions of dental and facial aspects that affect self-esteem and have significant impacts on OHRQoL item levels. 

## CONCLUSION

The convex and concave facial profile showed a negative impact on the psychological aspects of young adults’ quality of life.
